# Occurrence of hepatitis in an elderly woman during the treatment of pembrolizumab for right advanced renal pelvis, ureteral cancer, and bladder cancer

**DOI:** 10.1002/jgh3.12554

**Published:** 2021-05-06

**Authors:** Shinya Asatani, Tatsuo Kanda, Masayuki Honda, Tomotaka Ishii, Yoichiro Yamana, Tomohiro Kaneko, Taku Mizutani, Hiroshi Takahashi, Mariko Kumagawa, Reina Sasaki, Ryota Masuzaki, Shini Kanezawa, Naoki Matsumoto, Kazushige Nirei, Hiroaki Yamagami, Mitsuhiko Moriyama

**Affiliations:** ^1^ Division of Gastroenterology and Hepatology, Department of Medicine Nihon University School of Medicine Tokyo Japan

**Keywords:** hepatitis, immune‐related adverse event, pembrolizumab, renal pelvis and ureteral cancer, steroid

## Abstract

Recently, the use of immune checkpoint inhibitors (ICIs) with or without chemotherapeutic agents has been increasing in the treatment for advanced cancer. Here, we report the occurrence of liver failure after the use of pembrolizumab in an 82‐year‐old woman with metastatic liver disease derived from right advanced renal pelvis, ureteral cancer, and bladder cancer. She was successfully treated with 0.6 mg/kg daily prednisolone. In patients treated with ICIs, ICI‐induced hepatitis is occasionally observed. Even if patients are older, it appears important to diagnose and treat ICI‐induced hepatitis earlier by multidisciplinary therapies including steroid treatment. This is a first report of pembrolizumab‐induced liver failure in elder patient with age over 80 years. Even if patients are older, it appears important to diagnose and treat ICI‐induced hepatitis earlier by multidisciplinary therapies including steroid treatment.

## Graphical abstract

We experienced the occurrence of liver failure after the use of pembrolizumab in an 82‐year‐old woman with metastatic liver disease. Immune checkpoint inhibitor‐induced hepatitis is occasionally observed. It seems important to diagnose and treat older patients with immune checkpoint inhibitor‐induced hepatitis earlier by multidisciplinary therapies.

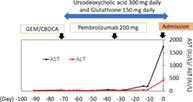

## Introduction

Treatments with immune checkpoint inhibitors (ICI) blocking cytotoxic T lymphocyte associated protein‐4 (CTLA‐4) and the programmed death‐1 (PD‐1) axis are common for advanced cancer.^1^ PD‐1 is a checkpoint that regulates the immune response. Pembrolizumab is a humanized monoclonal anti‐PD‐1 antibody that is effective in many cancer therapies. It is well known that patients with advanced urothelial carcinoma that progresses after platinum‐based chemotherapy have a poor prognosis.^2^ Pembrolizumab is associated with significantly longer overall survival and a lower rate of treatment‐related adverse events than chemotherapy as a second‐line therapy for platinum‐refractory advanced urothelial carcinoma.^2^ Pembrolizumab is one of the treatment options for patients with platinum‐refractory advanced urothelial cancer.^2^

Abnormality of liver function is occasionally observed during treatment with ICIs. Because ICIs are usually used for the patients with advanced cancer, metastatic liver disease, vascular liver disease accompanied with the use of anti‐vascular endothelial growth factor (VEGF) inhibitors, drug‐induced liver injury (DILI), exacerbation of viral hepatitis, autoimmune liver disease, ICI‐induced hepatitis, and metabolic liver disease may occur during ICI treatment.

We herein describe the occurrence of liver failure without encephalopathy as an immune‐related adverse event (irAE) in an elderly woman after receiving pembrolizumab for metastatic liver disease derived from right advanced renal pelvis, ureteral cancer and bladder cancer. In the presented patient with metastatic liver disease, ICI‐induced hepatitis caused liver failure as an acute insult. We successfully treated this patient with 0.6 mg/kg daily prednisolone.

## Case report

An 83‐year‐old female underwent perspective endoscopic nephroureterectomy; lymphadenectomy at the hilum of the kidney; and partial bladder resection for right advanced renal pelvis, ureteral cancer, and bladder cancer 10 months ago. The pathology report showed that the right renal pelvis and ureteral were invasive urothelial carcinoma and noninvasive high‐grade papillary urothelial carcinoma, respectively. She had relapsing urothelial carcinoma in the urinary bladder 6 months prior. She underwent posterior spinal fusion and received radiation therapy for spine metastasis 4 months ago.

Liver metastasis was observed 3 months ago. She initiated chemotherapy with the one‐cool combination of gemcitabine and carboplatin 2 months ago, and stopped this combination therapy due to the elevation of biliary enzyme and the judgment of progressive disease (PD) using Response Evaluation Criteria in Solid Tumors (RECIST) criteria. She received 200 mg pembrolizumab therapy 1 month ago. Due to liver failure with severe transaminase elevation and coagulopathy, she was admitted to our hospital. To our surprise, she was asymptomatic without complaint and was fully conscious and alert. Her body length, body weight, and body mass index were 1.48 m, 66.9 kg, and 30.7 kg/m^2^, respectively. Her body temperature, blood pressure, pulse rate, and blood oxygen saturation (at room air) were 36.4°C, 127/60 mmHg, 73 beat per minute, and 96%, respectively. She had no anemia, jaundice, or abnormal findings on chest or abdomen. She had slight edema and a scar from the operation on both legs and her back, respectively. Her performance status was Grade 2, according to the Eastern Cooperative Oncology Group (ECOG) performance status classification.

She had a history of cataract, hypertension, ovarian cyst, and boundary intelligence. She was not a drinker of alcohol or a smoker. Her family history was as follows: her father, cerebral infarction; her brother, gastric cancer; and her sister, uterine cancer. She had taken several drugs: amlodipine besilate (5 mg daily), lafutidine (20 mg daily), tramadol hydrochloride (37.5 mg/tablet) acetaminophen (325 mg/tablet) (four tablets daily), mirogabalin besilate (20 mg daily), celecoxib (200 mg daily), precipitated calcium carbonate, cholecalciferol, magnesium carbonate (two tablets daily), and furosemide (20 mg daily). She had also taken ursodeoxycholic acid (UDCA) 300 mg daily and glutathione 150 mg daily for biliary enzyme elevation due to the combination treatment of gemcitabine and carboplatin. She had no history of drug allergy.

Her laboratory data on admission to our department are shown in Table 1, Figure S1A, B. Marked elevation of serum transaminase and coagulopathy were noted. The chest X‐ray showed no lung metastasis. The electrocardiogram and echocardiogram showed no sign of heart diseases. Findings of computed tomography (CT) and abdominal ultrasound showed multiple liver metastases with mild ascites (Fig. S1C). Magnetic resonance cholangiopancreatography (MRCP) did not show dilatation of the bile duct (Fig. S1D). Therefore, the diagnosis was liver failure with liver metastasis and ICI‐induced hepatitis associated with the use of pembrolizumab as an acute insult.

**Table 1:** Laboratory data on admission (day 0) of the present case

Item	Value	Item	Value	Item	Value
WBC	**9.1 × 10^3^/μL**	AST	**1733 IU/L**	NH_3_	49 μg/dL
Hemoglobin	**9.7 g/dL**	ALT	**422 IU/L**	HBsAg	Negative
Platelets	178 × 10^3^/μL	LDH	**668 IU/L**	Anti‐HBs	**Positive**
Neutrophils	**70.1%**	ALP	**1754 IU/L**	Anti‐HBc	**Positive**
Basophils	0.7%	γ‐GTP	**275 IU/L**	HBV DNA	<1.0 LIU/mL
Eosinophils	1.8%	CPK	19 U/mL	Anti‐HCV	Negative
Monocytes	14.8%	Amylase	66 U/mL	IgM HAV	Negative
Lymphocytes	**12.6%**	T. Bil	1.66 mg/dL	IgA HEV	Negative
PT	**49%**	TP	**5.3 g/dL**	Anti‐HIV	Negative
INR	**1.55**	Albumin	**2.5 g/dL**	ANA	Negative
T. CHO	175 mg/dL	BUN	**25.3 mg/dL**	AMA M2	Negative
TG	99 mg/dL	Creatinine	0.92 mg/dL	IgG	1011 mg/dL
Glucose	100 mg/dL	eGFR	**44.2 mL/min/1.73m^2^**	IgA	240 mg/dL
HbA1c	5.8%	CRP	**8.62 mg/dL**	IgM	**47 mg/dL**

Values with non‐block indicate within normal limits.

γ‐GTP, γ‐glutamyl transpeptidase; ALP, alkaline phosphatase; ALT, alanine aminotransferase; AMA M2, antimitochondrial M2 antibody; ANA, antinuclear antibody; anti‐HBc, anti‐hepatitis B core antibody; anti‐HBs, anti‐hepatitis B surface antibody; anti‐HCV, anti‐hepatitis C virus antibody; AST, aspartate aminotransferase; BUN, blood urea nitrogen; CPK, creatinine phosphokinase; CRP, C‐reactive protein; eGFR, estimated glomerular filtration rate; HAV, hepatitis A virus; HbA1c, hemoglobin A1c; HBsAg, hepatitis B surface antigen; HEV, hepatitis E virus; HIV, human immunodeficiency virus; IgA, immunoglobulin A; IgG, immunoglobulin G; IgM, immunoglobulin M; INR, international normalized ratio; LDH, lactate dehydrogenase; NH_3_, ammonia; PT, prothrombin time; RBC, red blood cells; T. Bil, total bilirubin; T. CHO, total cholesterol; TG, triglyceride; TP, total protein; WBC, white blood cells.

As hepatic toxicity was Grade 3, according to the Common Terminology Criteria for Adverse Events (CTCAE) v5.0 classification, we stopped using pembrolizumab and administered 40 mg daily prednisolone. On day 22, aspartate aminotransferase (AST) and alanine aminotransferase (ALT) levels were improved. However, on day 37, she died due to primary disease.

## Discussion

It was reported that irAE development was associated with survival outcome of pembrolizumab treatment in patients with advanced urothelial cancer.^3^ Although high doses of corticosteroids were recommended for severe ICI‐induced hepatitis, the present case was an elderly woman with polypharmacy and a history of several diseases and metastatic liver diseases; therefore, we used the reduction‐doses of corticosteroids, and liver dysfunction was improved.

Tian *et al*. reported that ICI‐induced hepatitis is usually asymptomatic but may present with fever, malaise, and even death in rare cases.^4^ Our case was also asymptomatic, and her appetite was good upon admission with no fever. Diagnosis of ICI‐induced hepatitis is based on the exclusion of other etiologies according to the medical history, laboratory data, imaging, and liver biopsy findings.^4^ Treatment of ICI‐induced hepatitis requires discontinuation of ICI and/or treatment with immunosuppressants.^4^

We excluded vascular liver disease, viral hepatitis, or metabolic liver disease as a cause of liver dysfunction due to the history and laboratory data. Response to corticosteroids could exclude liver dysfunction due to metastatic liver diseases. It is difficult to differentially diagnose autoimmune hepatitis, DILI, or ICI‐induced hepatitis. We did not perform liver biopsy due to the coagulopathy. We ruled out classical autoimmune hepatitis from serum immunoglobulin levels and negative results for antinuclear antibodies. DILI, caused by other drugs, was also ruled out from the clinical course.

There are varied opinions on the effects of steroids on antitumor responses. Horvat *et al*. reported that 35% of melanoma patients treated with ipilimumab required systemic corticosteroid treatment for irAEs and that there was no difference in overall survival or time to treatment failure between patients with and without the administration of systemic corticosteroids to treat an irAE.^5^ However, high‐dose glucocorticoids for the treatment of ipilimumab‐induced hypophysitis was associated with reduced survival in melanoma patients.^6^ Careful attention should be paid to the use of long‐term steroids in patients with advanced malignancies.

Imoto *et al*. reported that 56 of 387 (16.3%) patients treated with ICIs for nonhepatic malignancy experienced liver dysfunction.^7^ They also reported that 11 of these 56 (19.6%) patients processed Grade 3 or 4 hepatitis, resembling autoimmune hepatitis with normal IgG levels, and 2/11 (9.1%) were positive for antinuclear antibody. In patients treated with ICIs and equal to or more than the Grade 3 liver dysfunction, the permanent discontinuation of ICIs and the use of 1–2 mg daily corticosteroids were recommended.^8^ We reduced the dose to 0.6 mg/kg daily prednisolone for treatment of the present case.

The present case seemed the first report of pembrolizumab‐induced liver failure in elder patient with age over 80 years, although ICI‐induced hepatitis cases were often seen among patients treated with pembrolizumab. Sakakida *et al*. reported the occurrence of fatal fulminant hepatic failure after the use of nivolumab for an 80‐year‐old woman with advanced renal cell carcinoma.^9^ Special attentions should be paid to their liver function during the treatment of ICI for elder patients. In conclusion, we experienced the occurrence of liver failure after the use of pembrolizumab for an 82‐year‐old woman with metastatic liver disease derived from right advanced renal pelvis, ureteral cancer, and bladder cancer. In the cases treated with ICIs, ICI‐induced hepatitis is not rare. Even if cases are in the elderly, it is important to diagnose and treat them earlier by multidisciplinary therapies, including steroid treatment.

## Supporting information


**Figure S1.** Clinical courses and findings of abdominal computed tomography (CT) and magnetic resonance cholangiopancreatography (MRCP) examination at admission. (A) Acute exacerbation of ALT level was observed 1 month after the administration of 2000 mg pembrolizumab. GEM: gemcitabine (1000 mg), CBDCA: carboplatin (450 mg). (B) The patient initiated treatment with prednisolone (PSL) 40 mg daily with stronger neo‐minophagen C (SNMC), ursodeoxycholic acid (UDCA) and vitamin K. On day 37 after admission, she died due to primary disease. (C) Contrast‐enhanced CT showed multiple metastatic tumors in the liver and mild ascites on the liver surface. (D) MRCP also showed multiple metastatic tumors in the liver and mild ascites on the liver surface. No dilatation of the bile duct was observed.
