# Supplementary material for: Occurrence of hepatitis in an elderly woman during the treatment of pembrolizumab for right advanced renal pelvis, ureteral cancer, and bladder cancer
Source: JGH Open. 2021 May 6;5(6):722–4. doi: 10.1002/jgh3.12554 (PMC8171158; doi:10.1002/jgh3.12554)
Supplement: Supplementary file 1 — Figure S1. Clinical courses and findings of abdominal computed tomography (CT) and magnetic resonance cholangiopancreatography (MRCP) examination at admission. (A) Acute exacerbation of ALT level was observed 1 month after the administration of 2000 mg pembrolizumab. GEM: gemcitabine (1000 mg), CBDCA: carboplatin (450 mg). (B) The patient initiated treatment with prednisolone (PSL) 40 mg daily with stronger neo‐minophagen C (SNMC), ursodeoxycholic acid (UDCA) and vitamin K. On day 37 after admission, she died due to primary disease. (C) Contrast‐enhanced CT showed multiple metastatic tumors in the liver and mild ascites on the liver surface. (D) MRCP also showed multiple metastatic tumors in the liver and mild ascites on the liver surface. No dilatation of the bile duct was observed. [file JGH3-5-722-s001.docx]

**Supporting Figure legends**

Figure S1. Clinical courses and findings of abdominal computed tomography (CT) and magnetic resonance cholangiopancreatography (MRCP) examination at admission.

(A) Acute exacerbation of ALT level was observed one month after the administration of 2000 mg pembrolizumab. GEM: gemcitabine (1000 mg), CBDCA: carboplatin (450 mg). (B) The patient initiated treatment with prednisolone (PSL) 40 mg daily with stronger neo-minophagen C (SNMC), ursodeoxycholic acid (UDCA) and vitamin K. On day 37 after admission, she died due to primary disease. (C) Contrast-enhanced CT showed multiple metastatic tumors in the liver and mild ascites on the liver surface. (D) MRCP also showed multiple metastatic tumors in the liver and mild ascites on the liver surface. No dilatation of the bile duct was observed.


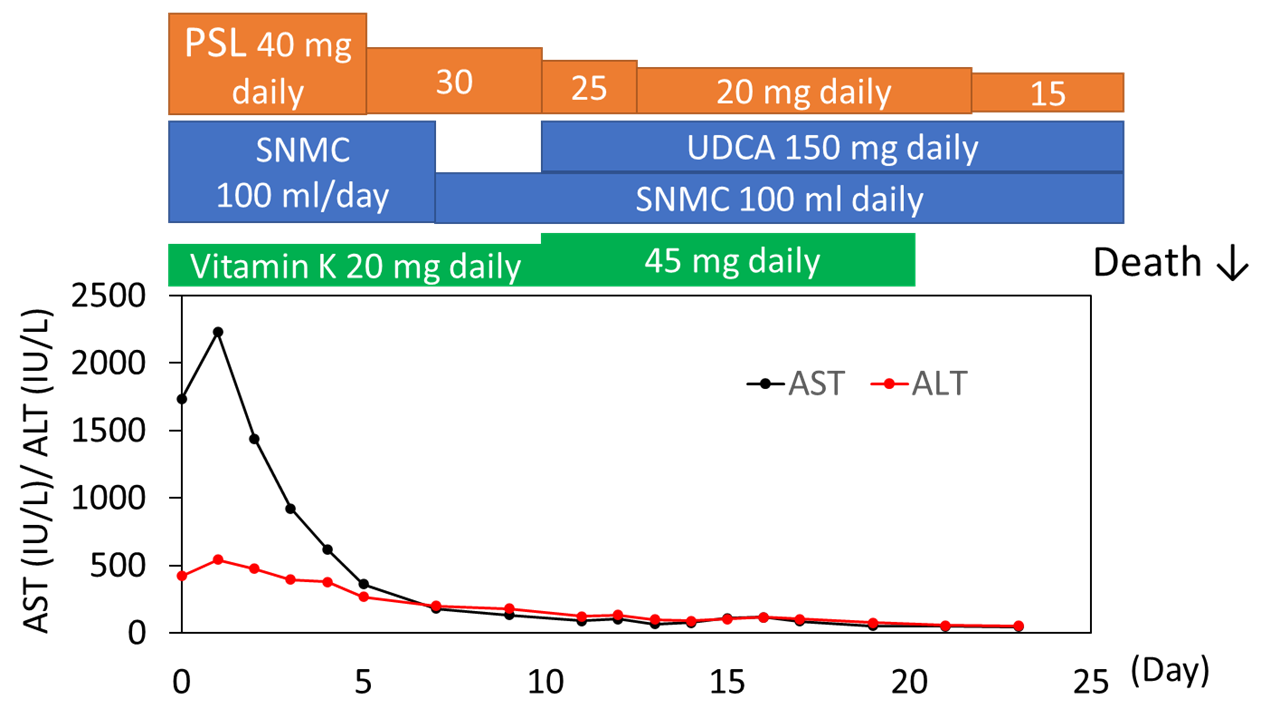


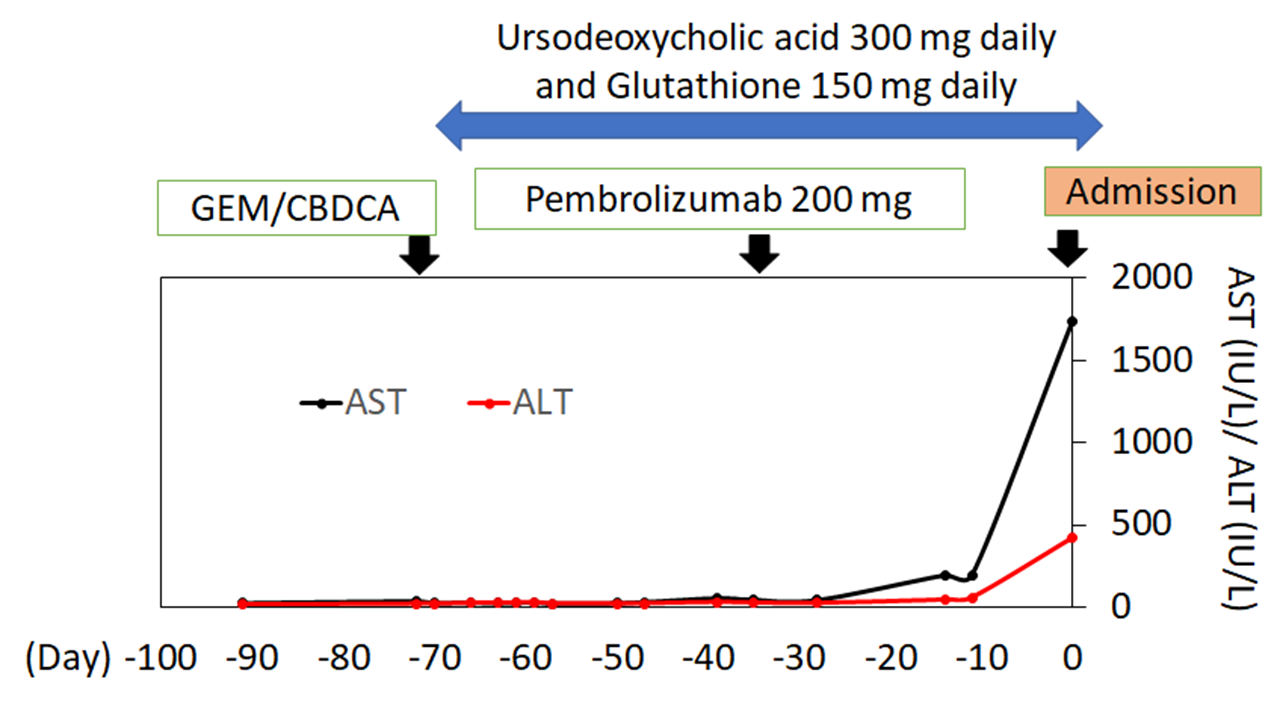


**Fig. S1 (A)**

**Fig. S1 (B)**


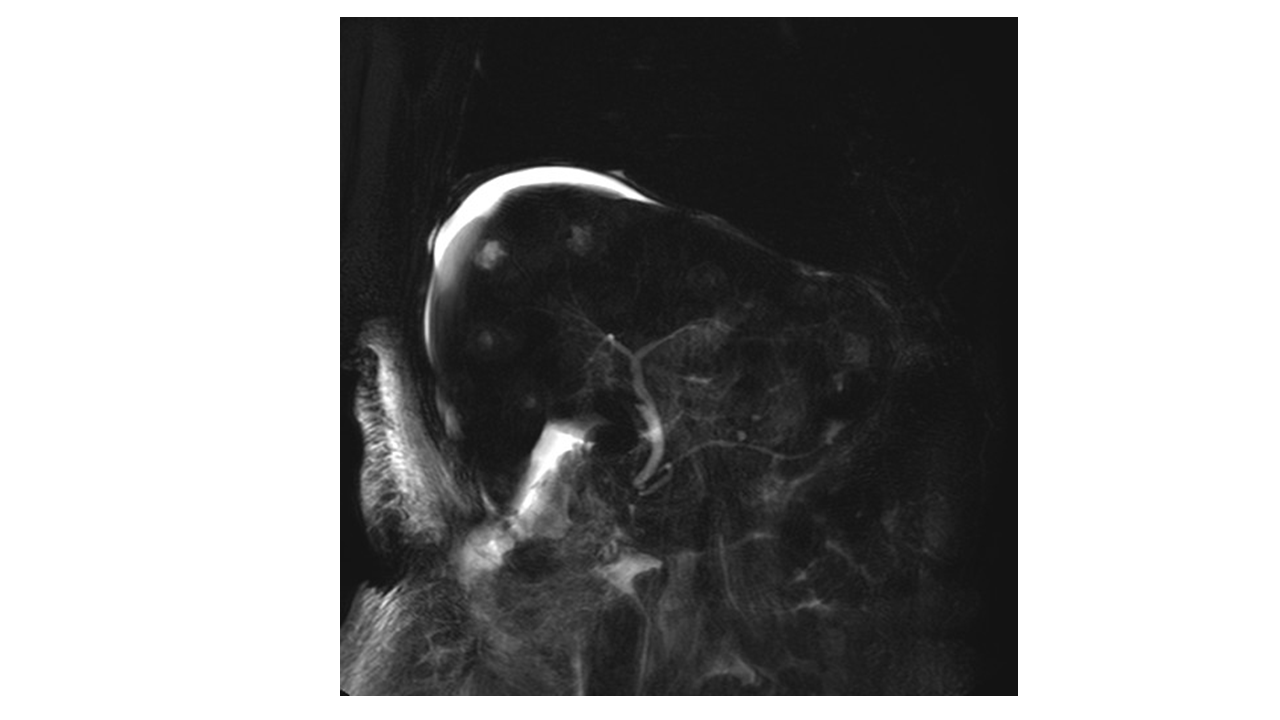


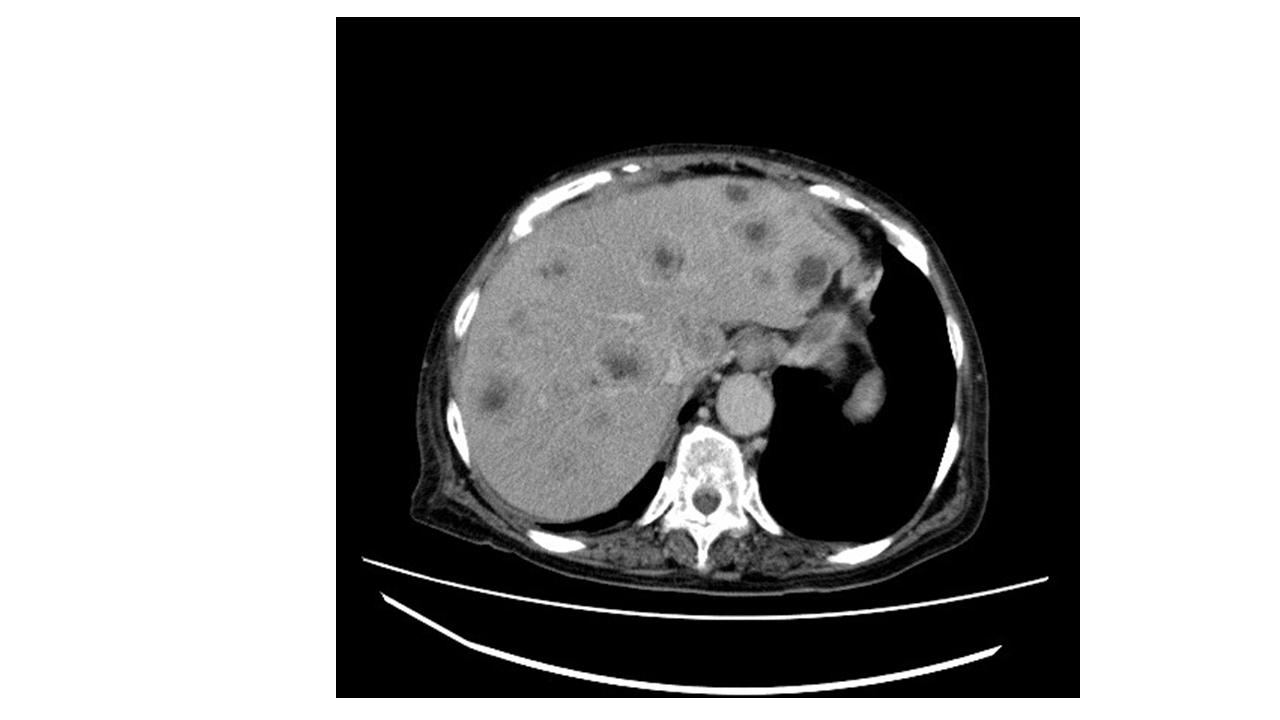


**Fig. S1 (C)**

**Fig. S1 (D)**
